# Machine Learning-Driven E-Nose-Based Diabetes Detection: Sensor Selection and Feature Reduction Study

**DOI:** 10.3390/s25216607

**Published:** 2025-10-27

**Authors:** Yavuz Selim Taspinar

**Affiliations:** Department of Mechatronic Engineering, Selcuk University, Konya 42130, Turkiye; ytaspinar@selcuk.edu.tr; Tel.: +90-533-5676297

**Keywords:** electronic nose, machine learning, feature reduction, rank analysis, feature analysis

## Abstract

**Highlights:**

**What are the main findings?**

- The Artificial Neural Network (ANN) model achieved the best performance, reaching 100% accuracy in diabetes classification using e-nose data.
- Feature selection via ANOVA and Information Gain identified TGS2610 and TGS2611 sensors as the most discriminative for diabetes detection.

**What is the implication of the main finding?**

- Optimized sensor selection and dimensionality reduction enable faster and more efficient model training without compromising accuracy.
- The proposed e-nose-based machine learning framework supports the development of non-invasive, practical, and clinically applicable diagnostic tools for diabetes.

**Abstract:**

Diabetes is a major global health problem, with a rapidly increasing prevalence and long-term health complications in both developed and developing countries. If not diagnosed early, it can lead to cardiovascular diseases, kidney failure, vision loss, and nervous system disorders. This study aimed to classify individuals with diabetes or healthy individuals using e-nose sensor data obtained from breath samples taken from 1000 individuals. Six sensor features and one class feature were used in the analysis. Machine learning methods included Artificial Neural Networks (ANN), Decision Trees (DT), Gradient Boosting (GB), Naive Bayes (NB), and AdaBoost (AB). ANOVA and Information Gain analyses were conducted to determine the effectiveness of the sensor data, and the TGS2610 and TGS2611 sensors were found to be critical for classification. Principal Component Analysis (PCA) reduced data size and saved processing time. Experimental results showed that the ANN model provided the most successful classification, with 100% accuracy. AB and GB achieved 99.8% accuracy, while NB achieved 97.6% accuracy. Dimensionality reduction using PCA optimized training and testing times without loss of accuracy. The study presents a data-driven approach to e-nose-based diabetes detection, demonstrates the comparative performance of the models, and highlights the importance of sensor selection and data size optimization.

## 1. Introduction

Diabetes has become a global health problem today, with its rapidly increasing prevalence in both developed and developing countries, long-term health complications, and societal costs [1]. According to World Health Organization data, if not diagnosed early, diabetes can lead to serious problems such as cardiovascular disease, kidney failure, vision loss, and nervous system disorders [2]. Therefore, early detection of the disease is a critical step that directly impacts the success of treatment. While traditional diagnostic methods often rely on invasive procedures such as blood tests, in recent years, faster, easier, and non-invasive alternative approaches have begun to emerge thanks to developing sensor technologies and data analysis methods. In particular, the evaluation of biomarkers obtained from individuals’ breath has attracted attention as a promising method for diagnosing metabolic diseases such as diabetes [3]. Analyzing such biomedical data alone is often limited, but when supported by advanced artificial intelligence and machine learning algorithms, highly accurate classifications are possible. Machine learning plays a crucial role in extracting meaningful patterns from large and complex datasets, determining the importance of features, and developing decision support systems that contribute to disease diagnosis [4]. Recent studies have shown that processing sensor-based data with different algorithms not only achieves high success rates but also reveals which features are most effective in classification. Furthermore, making data more understandable and processable through dimensionality reduction techniques and statistical analysis methods not only increases classification accuracy but also offers practical solutions that can be used in clinical applications. In this context, analyzing sensor-based data for diabetes detection using machine learning methods constitutes a current and innovative research topic that supports diagnostic processes in the healthcare field and demonstrates the clinical utility of artificial intelligence applications [5]. In recent years, a remarkable number of innovative research and applications have been developed in the literature in these areas. These studies demonstrate that the subject is undergoing rapid development at both theoretical and industrial levels.

In their study, Wu et al. performed gas classification using Transformer Encoder (TE) and Temporal Convolutional Network (TCN)-based methods using gas data obtained from the e-nose system. They achieved 99.8% accuracy results using the TETCN model and Bayesian parameter optimization methods. They developed a gas detection device. It has been used in time series analysis and gas detection applications. They proposed a hybrid model and a new method that provides high accuracy in the literature [6].

Chen et al. performed variety classification and origin determination using SACNet-based methods and the e-nose system using gas data obtained from pepper samples of different varieties and origins. They achieved 98.56% variety classification, 97.43% origin identification, and 99.31% overall accuracy results using the SACNet model and sensor attention module methods. They developed an e-nose-based pepper recognition device. It has been used in agricultural product quality control and food safety. They have made contributions to the literature such as a high-accuracy classification model and a sensor-aware approach [7].

Kombo et al. In their study, they performed quality classification of black tea using black tea gas data obtained from (Metal Oxide Semiconductor Sensors) MOS-based e-nose system using piecewise feature extraction, PCA, Linear Discriminant Analysis (LDA) and Support Vector Machine (SVM) methods. They achieved 99.50% training, 95.30% validation and 96.50% test accuracy with 98.60% sensitivity and 99.10% specificity results from the SVM model and piecewise feature extraction method. They developed the e-nose device for quality determination of black tea. It has been used in the field of food quality control and agricultural product evaluation. They presented an e-nose-based quality classification approach that provides high accuracy in the literature [8].

In their study, Karami and Kroshrou used e-nose data from essential oils to classify them using GBLinear and TabNet algorithms and feature selection methods. They achieved high accuracy and interpretability results from the GBLinear and TabNet-based hybrid methods. They developed an e-nose-based device for the classification of essential oils. It has been used in quality control in the food, cosmetics, and pharmaceutical industries. They presented a new method and interpretable machine learning techniques for the analysis of complex gas mixtures [9].

Moline et al. used e-nose data from chocolate samples to perform single-label and multi-label classifications using the Artificial Immune System (AIS)-based method. They achieved 97% accuracy in single-label classification and 94% accuracy in multi-label classification using the AIS algorithm. They developed an AIS-based e-nose device for identifying chocolate varieties. It has been used in food quality control and product variety analysis. They proposed that chocolate samples could be identified by adapting the multi-label learning approach to e-nose systems and using alternative classification algorithms [10].

Divyashree et al. performed gas type classification and concentration estimation using e-nose data with four nanosensors. They obtained gas type classification results using PCA and SVC (with RBF kernel) methods and concentration estimation results using the AdaBoost method. The system has been used in the fields of gas analysis and sensor technologies. They have made contributions to the literature such as improving the performance of electronic noses for gas recognition and concentration estimation using machine learning [11].

Kakaei et al. used e-nose data to detect and classify counterfeit in some fossil fuel products such as gasoline, diesel, and kerosene. They obtained fuel type classification results using LDA, Quadratic Discriminant Analysis (QDA), SVM, and ANN methods. They developed an e-nose device with 10 MOS sensors. This system has been used in fuel analysis and counterfeit detection. They have contributed to the literature by demonstrating that electronic noses can detect fossil fuel counterfeits with high accuracy and improve classification performance [12].

Jiang et al., in their study, used e-nose data to perform gas type classification and concentration estimation. They obtained gas classification and concentration estimation results using the Time–Frequency Attention Convolutional Neural Network (TFA-CNN) method. They developed the e-nose device using two types of metal oxide semiconductor gas sensors. This system has been used in gas analysis and environmental monitoring. They have contributed to the literature by presenting a deep learning-based method that effectively utilizes time-frequency information in e-nose signals, improving the performance of gas classification and concentration estimation [13].

Du et al. used e-nose data to classify mixed gases. They obtained gas classification results by processing sensor array signals from the Vision Transformer (ViT) model with all its processes. The model classified acetone, methanol, ammonia, and their binary mixtures with 96.66% accuracy; for comparison, SVM and 1D CNN models showed 90.56% and 92.75% accuracy, respectively. This method improves performance with a self-attention mechanism that combines global information and enables adaptive feature extraction. They developed the e-nose device for mixed gas analysis. They contributed to the literature on improving the accuracy of mixed gas classification by effectively utilizing multi-channel time series data [14].

Tand and Wang used e-nose data to perform quality determination during rice storage. They obtained gas classification results using the Multi-branch Self-attention Module (MSAM) and MSAM-Net methods. They developed the e-nose device with a PEN3 sensor. This system has been used in rice quality monitoring and food safety. At 25 °C and 35% RH, 96.25% accuracy and an F1-score of 96.84% were achieved, and at 40 °C and 35% RH, 97.42% accuracy and an F1-score of 97.64% were achieved. They have contributed to the literature by combining artificial intelligence with gas sensors to rapidly and accurately determine rice quality depending on storage conditions [15].

Pu et al. used e-nose data to identify characteristic gases released during thermal runaway in lithium batteries. They obtained gas classification and concentration estimation results using PCA, MultiLayer Perception (MLP), Extreme Learning Machine (ELM), and SVM methods. They developed an e-nose device with three MOS sensors. This system has been used in lithium battery safety and gas analysis. The MLP classification algorithm achieved 99.23% accuracy with a 20 s response time. In concentration regression, when classification results and raw features were combined and used with multi-output MLP, the RMSE was significantly reduced. They contributed to the literature by accurately classifying characteristic gases in a short time and estimating their concentrations, thus improving battery safety monitoring performance [16].

Shtepliuk et al. used e-nose data in their study to perform post-mortem inspection and quality control of pork. They obtained freshness and contamination classification results from Optimizable Ensemble and other Machine Learning (ML) methods. They developed the e-nose device with a metal oxide sensor array. This system has been used in meat inspection and food safety. The model distinguished between fresh and urine-contaminated meat samples with 96.5% sensitivity and 95.3% specificity; it also classified meat aged for one to two days with 93.5% accuracy. They suggested that the combination of e-nose and artificial intelligence could enable high-accuracy meat freshness and contamination determination and streamline meat inspection processes [17].

Ni et al. performed gas type classification and concentration estimation using e-nose data. They obtained classification and regression results using Multi-Task Learning (MTL), Convolutional Neural Network (CNN), and Long Short-Term Memory (LSTM)-based methods. They developed the e-nose device using an autonomous gas detection test system and a Gramian Angular Field-Markov Transition Field transform. This system has been used in gas analysis and electronic nose technologies. Despite compressing the images by 3.9%, the model achieved 95.31% accuracy in classification and an R^2^ score of 0.9510 in regression. They suggested that deep learning-based multitasking models improve e-nose performance by integrating temporal and spatial features [18].

Binson et al. performed lung cancer detection using e-nose data. They obtained VOC gas classification results using LDA and other ML methods. They developed the e-nose device with five VOC gas sensors. This system has been used in medical electronics and respiratory analysis. The LDA algorithm achieved 93.14% accuracy, 88.63% sensitivity, and 95.62% specificity; the Area Under Curve (AUC) value was 0.98. They suggested that lung cancer could be detected by analyzing VOCs in human breath with a non-invasive, low-cost, and fast-response e-nose [19].

Xia et al. conducted tea quality classification using e-nose and Near-Infrared Spectroscopy (NIRS) data in their study. They obtained taste and aroma quality classification and content estimation results from SVM, K Nearest Neighbor (KNN), and ANN methods. They developed the system by integrating e-nose and NIRS devices. This system has been used in tea production and quality control. NIRS alone achieved 99.63% accuracy, and e-nose achieved 97.00% accuracy. With data fusion, SVM achieved 98.13% accuracy, KNN 96.63% accuracy, and ANN 97.75% accuracy. They suggested that tea quality could be determined quickly and with high accuracy with the integration of NIRS and e-nose [20].

Govindarajan et al. used e-nose data to classify gases in transformer oil. They obtained classification results for H_2_, CH_4_, C2H_6_, C_2_H_4_, and C_2_H_2_ gases using different machine learning methods. They developed the e-nose device with a metal-oxide semiconductor sensor array. This system was used in transformer oil condition and electrical equipment monitoring. After feature ranking and dimensionality reduction, the F1 score reached 0.9313, and after sensor selection with SVM and genetic algorithm, it reached 0.9869. The MQ8 and TGS2612 sensors showed the best performance. They suggested that a combination of e-nose and machine learning could provide fast, low-cost, and highly accurate transformer oil gas analysis [21].

Sun et al. used e-nose data to perform soybean quality monitoring. They obtained gas classification results from AKCA-Net and Adaptive Convolutional Kernel Channel Attention (AKCA) methods. They developed the e-nose device to collect soybean gas information from different origins. This system has been used in agricultural product quality monitoring and counterfeiting. AKCA-Net demonstrated 98.21% accuracy, 98.57% sensitivity, and 98.60% sensitivity. They contributed to the literature by combining e-nose and a deep learning-based attention mechanism with high-accuracy soybean quality monitoring and determining its origin [22].

Mirda et al. conducted edible oil quality classification using e-nose data. They obtained classification results for fresh and bleached waste oil using Random Forest and other machine learning methods. They developed the e-nose device to collect gas and odor data for fresh and processed oils. This system has been used in food safety and oil quality monitoring. The Random Forest algorithm achieved 99.47% accuracy, 99.50% sensitivity, 99.47% sensitivity, and 99.48% F1 score. They suggested that the combination of e-nose and machine learning could ensure high accuracy separation of fresh and bleached waste oil and protect consumer health [23]. Gudiño-Ochoa et al. performed diabetes detection using e-nose data. They obtained diabetes classification results based on acetone in human breath using XGBoost, Deep Neural Network (DNN), and 1D-CNN methods. They developed the e-nose device with a MOS sensor and a TinyML-based embedded system. This system has been used in medical diagnosis and non-invasive diabetes detection. The XGBoost algorithm achieved 95% accuracy, while deep learning-based models achieved 94.44% accuracy. They suggested that real-time, non-invasive, and highly accurate diabetes detection can be achieved by integrating e-nose and TinyML in embedded systems [24].

Gudiño-Ochoa et al. used e-nose data to perform diabetes detection and Blood Glucose Level (BGL) estimation. They obtained BGL estimation and diabetes classification results using breath VOCs using LightGBM (Light Gradient Boosting Machine) and Random Forest methods. They developed the e-nose device with a MOS sensor using a TinyML-based embedded system. This system has been used in non-invasive diabetes monitoring and medical diagnosis. BGL estimation was achieved with 86% accuracy with the LightGBM model, and diabetes detection was achieved with 94.14% accuracy with Random Forest. They have made contributions to the literature, such as providing real-time, non-invasive, and highly accurate diabetes monitoring with TinyML-based e-nose using real and synthetic data [25].

Vadera and Dhanekar classified VOCs in human breath using e-nose data. They obtained classification results of breath samples from Extra Trees and other ML algorithms. They developed the e-nose device equipped with gas sensors into an IoT-based portable system. This system has been used in breath analysis and medical diagnosis. The model classified normal breath, alcohol (simulated breath), mint, mouthwash, and cardamom samples with 96% accuracy. They have made contributions to the literature, such as presenting a non-invasive approach that provides high accuracy in disease and physiological state detection through breath, by combining IoT-integrated e-nose and machine learning [26].

Gudiño-Ochoa et al. classified diabetes using e-nose data. They obtained classification results for breath samples using Random Forest, Gradient Boosting, and their ensemble models. They developed the e-nose device with three gas sensors sensitive to CO, alcohol, and acetone. This system has been used in non-invasive diabetes screening and health monitoring. The ensemble model achieved 98.86% accuracy, 99.07% sensitivity, 98.81% sensitivity, and 98.87% F1 score. They suggested that gas sensor-based breath analysis can provide fast, highly accurate, and scalable diabetes detection [27]. Beatriz Méndez-Rodríguez et al. used e-nose data to determine the risk of chronic kidney disease (CKD) after preeclampsia. They obtained classification results for urine metabolites using Canonical Analysis of Principal Coordinates (CAP) and PERMANOVA. They developed the e-nose device with the Cyranose^®^ 320 sensor for urine sample analysis. This system has been used in pregnancy monitoring and early diagnosis of kidney disease. The CAP model achieved 68.4% accuracy between the groups, and a statistically significant difference was found (*p* = 0.0001). They stated that the method they proposed allows early detection of CKD risk after preeclampsia using metabolomic patterns in urine [28].

Ozsandikcioglu et al. performed lung cancer detection using e-nose data. They obtained classification results of breath samples using PCA and LDA methods. They developed a hybrid sensor e-nose device with eight MOS and 14 Quartz Crystal Microbalance (QCM) sensors. This system has been used in respiratory analysis and medical diagnosis. Accuracy was 81.54% with metal oxide sensors and 73.18% with QCM sensors; when sensor data was combined, accuracy increased to 85.26%. The highest accuracy was 94.58% with LDA and 88.56% with PCA. They stated that high-accuracy detection of lung cancer from breath can be achieved with multi-sensor integration and dimensionality reduction methods [29].

Durán et al. performed colorectal cancer (CRC) detection using e-nose data. They obtained classification results of breath samples using PCA, Logistic Regression (LR), and other supervised learning methods. They developed the e-nose device, which features a 14-sensor MEMS (Micro-Electro-Mechanical Systems) metal oxide gas sensor array. This system has been used for the non-invasive and rapid detection of colorectal cancer. The PCA + LR model achieved 91.9% accuracy, 92.3% sensitivity, and 94.3% specificity. They contributed to the literature by providing a portable, low-cost, and effective method for detecting CRC through breath analysis [30].

This study was planned based on these studies in the literature. The procedures performed in the study are as follows:

- A dataset containing six features and one class feature was used to classify diabetic and healthy individuals based on breath samples taken from 1000 individuals using different sensors.
- Feature importance analysis was performed on the sensor data using ANOVA and Information Gain methods, and the most effective sensors and measurements were determined.
- Classification models were created using ANN, DT, GB, NB, and AB methods. Performance comparisons were conducted on all sensor data, dimensionally reduced features, and selected features.
- Classification processes were repeated on lower-dimensional data by reducing the data dimensionality using PCA. Correlation analysis and frequency distributions between sensors were visualized.
- The models’ accuracy, precision, sensitivity, and F1 scores were calculated, and the classification results obtained with dimensionally reduced features and selected features were compared with the results obtained with all features.

As a result of these studies, the contributions of this study to the literature are as follows:

- A scalable study on e-nose-based diabetes detection was presented using a large dataset of 1000 people.
- A data-based guide for sensor selection and optimization was provided by determining which sensors were more effective in classification using ANOVA and Information Gain analysis.
- By comparing the performance of different classification algorithms on the same dataset, a model comparison and best method recommendations were presented in the literature.
- Dimensionality reduction and classification performance analysis using PCA contributed to optimizing data efficiency and processing time.
- A more in-depth data analysis approach for e-nose-based diabetes detection was presented by identifying relationships between sensors and examining and interpreting frequency distributions.

The article is structured as follows: The Introduction summarizes the study motivation and literature. The Materials and Methods section describes data collection, sensors, data preprocessing, feature selection, and machine learning methods. The Experimental Results section presents classification performance and comparisons. The Discussion and Conclusion section provides interpretations of the results and their potential for application, summarizes their contributions, and offers suggestions for future work.

## 2. Materials and Methods

This section describes in detail the e-nose dataset, various machine learning methods, and classification algorithms used in this study. We also discuss dimensionality reduction techniques, feature selection methods, and cross-validation strategies used to improve classification performance and model interpretability. The confusion matrix and performance metrics used in the model evaluation process are also presented in this section.

### 2.1. E-Nose Sensor Dataset for Predicting Human Diseases

**Figure 1:**
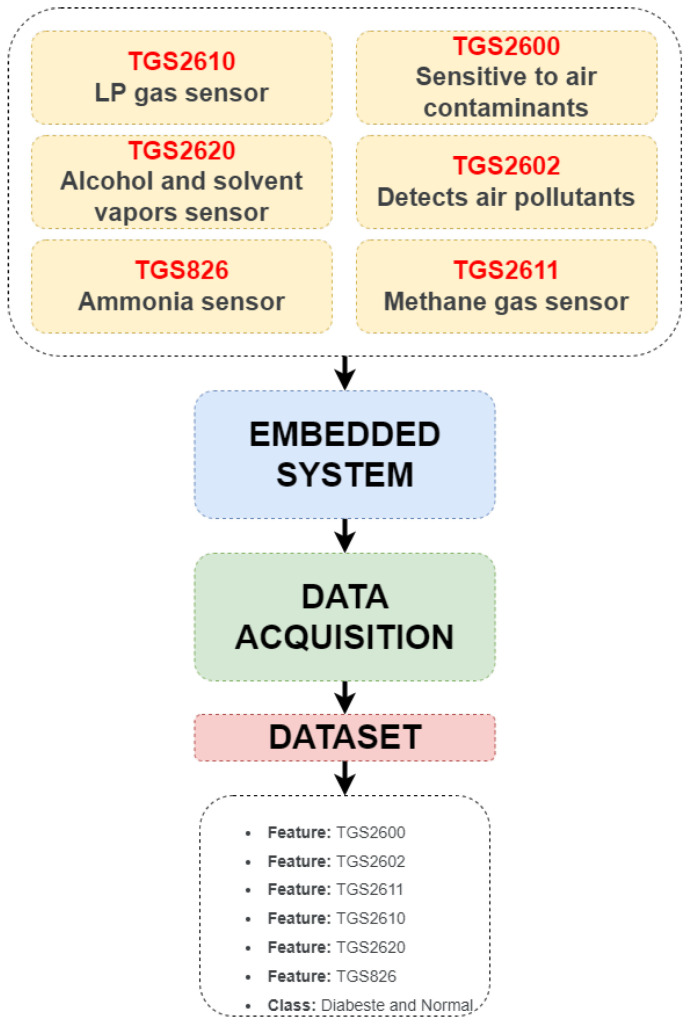
Dataset creation stages.

The dataset used in the study is the “e-nose Sensor Dataset for Predicting Human Diseases.” It was downloaded from kaggle.com [31]. The dataset contains data obtained from various gas sensors. There are a total of seven features: six features and one class feature. Data from 1000 individuals totals 1000 rows of data. No normalization or preprocessing was performed on the dataset. Data were collected from both diabetic and non-diabetic patients. There is no information in the dataset description regarding the time period for which data were collected from each patient. The dataset contains data from 545 diabetic and 455 normal individuals. It can be said that the distribution of data between classes is balanced. The sensors used in the dataset and the stages of its creation are shown in Figure 1.

### 2.2. Machine Learning Methods

#### 2.2.1. Decision Tree (DT)

Decision trees (DTs), frequently used in classification problems, stand out, particularly in the healthcare field, due to their simple structure and high interpretability. Essentially, data are divided into branches based on specific characteristics, and a hierarchical structure is created by selecting the most appropriate discrimination criterion (e.g., Information Gain or Gini index) at each node. This method allows complex datasets to be reduced to more understandable rules. In the detection of diabetes with e-nose systems, sensor responses to volatile organic compounds obtained from breath samples can be classified using decision trees. For example, a high response from a particular sensor can be considered a critical node in distinguishing individuals with diabetes. Thus, the system provides easily interpretable decision paths for individuals with diabetes and those with healthy behavior. However, decision trees can lead to overfitting problems when they have excessive branches, weakening their generalization ability. Therefore, pruning techniques or ensemble methods such as AdaBoost and Random Forest are recommended. As a result, decision trees are considered an important method in e-nose-based diabetes detection due to both their fast processing capacity and their ability to produce clinically explainable decision rules [32].

#### 2.2.2. AdaBoost (AB)

The use of AdaBoost is particularly advantageous in medical classification problems such as diabetes detection. This method produces more reliable results by combining classifiers that are not powerful enough on their own in high-dimensional and complex sensor data. Breath samples obtained from electronic nose (e-nose) systems can often contain noise due to biological variability and environmental factors. AdaBoost assigns more weight to misclassified samples, allowing subsequent classifiers to focus on this critical data, thus increasing sensitivity in distinguishing between diabetic and healthy individuals. Furthermore, AdaBoost maintains generalization ability by keeping the risk of overfitting relatively low, increasing reliability in clinical applications. Thanks to the adaptive weighting provided by the method, it is possible to more effectively evaluate small but significant biomarkers (volatile organic compounds) in breath data. Therefore, AdaBoost makes a strong contribution to e-nose-based machine learning studies in the early diagnosis of diabetes and offers a fast and highly accurate classification mechanism [33].

#### 2.2.3. Artificial Neural Network (ANN)

The ANN is one of the machine learning methods inspired by biological neural systems. ANNs consist of artificial neurons arranged in layers, and each neuron weights input data and converts it into output through activation functions. This structure allows learning complex nonlinear relationships and achieves high performance in many problems such as classification, regression, and pattern recognition [34]. ANNs can learn meaningful patterns from high-dimensional and noisy data coming from sensors in e-nose sensor data used for diabetes detection. Multilayer structures, in particular, offer a powerful approach for distinguishing diabetic and healthy individuals by modeling nonlinear relationships between sensor data. Furthermore, ANNs can adapt to the dataset by continuously updating the weights during the learning process, and their generalization ability can be increased through cross-validation. In this context, it is envisaged that ANN-based models can yield significant inferences in e-nose-based diabetes detection studies [35].

#### 2.2.4. Gradient Boosting (GB)

GB is an ensemble method that creates strong prediction models by sequentially combining weak classifiers (usually decision trees). The GB algorithm focuses on minimizing the residual error of the previous model at each iteration, thereby gradually increasing the model’s predictive power. Essentially, gradient descent optimization minimizes the loss function and directs the learning process in a controlled manner. In e-nose sensor data for diabetes detection, the GB algorithm is particularly capable of capturing complex and nonlinear data distributions and achieving high classification accuracy thanks to small error corrections. Furthermore, careful selection of its parameters (learning rate, tree depth, and number of iterations) reduces the risk of overfitting the model and increases its generalization ability. Therefore, the GB algorithm stands out as a strong candidate method for e-nose-based diabetes detection [36].

#### 2.2.5. Naive Bayes (NB)

NB is one of the probability-based classification algorithms that can achieve high performance despite its simple structure. Working based on Bayes’ theorem, the NB algorithm evaluates conditional probability relationships between classes and assumes that each feature is independent of the other. While this independence assumption is not fully met in most real datasets, the algorithm can produce quite successful results in practice. In breath data obtained from e-nose sensors for diabetes detection, NB can perform fast and reliable classification, especially in high-dimensional and noisy data environments. The advantages of the NB algorithm include low computational cost, easy applicability, and satisfactory accuracy even in small datasets. However, performance may decrease in cases where there are strong dependencies between features. Despite this, the NB algorithm is frequently preferred as the primary comparison method for e-nose-based diabetes diagnosis [37].

### 2.3. PCA for Feature Reduction (PCA)

PCA is a powerful statistical technique that performs dimensionality reduction while preserving the information density in high-dimensional data. PCA considers correlations between original variables and transforms these variables into new components (principal components) through linear combinations. These components represent a large portion of the total variance in the data and allow classification operations to be performed more quickly and efficiently with fewer dimensions. In diabetes detection data obtained from e-nose sensors, the high multidimensionality and high correlation of sensor measurements make PCA crucial. PCA reduces noise and redundant information, thereby improving the accuracy and generalization performance of machine learning algorithms. Furthermore, models operating in lower-dimensional data spaces reduce processing time and can provide more efficient solutions in hardware-constrained environments such as embedded systems [38].

### 2.4. Analysis of Variance ANOVA

ANOVA is a method that evaluates statistically significant differences in mean values between different groups. When diagnosing diabetes using e-nose data, it is critical to determine which features contribute more to the classification process among the multidimensional measurements obtained from sensors. In this regard, ANOVA analyzes the variance of sensor responses in breath samples obtained from diabetic and healthy individuals, allowing the selection of sensors or measurement features that show significant differences. This way, only the most discriminatory and effective sensor data is fed into the classification algorithms. ANOVA is also used to reduce model complexity and minimize the effect of noise. Especially in e-nose data containing numerous sensors and variables, eliminating low-impact or redundant features increases the accuracy and generalization ability of machine learning models. This method reduces unnecessary data load during the model training process and ensures more reliable classification performance. Furthermore, ANOVA results provide guidance for optimizing sensor design and the e-nose system. It supports future sensor selection and placement strategies by identifying which sensors are more critical [39].

### 2.5. Information Gain (IG)

Information Gain is an effective metric used in classification problems to determine the extent to which features explain the target variable. When detecting diabetes using e-nose data, it is crucial to determine which features of sensor responses obtained from breath samples are more decisive in classifying individuals as diabetic or healthy. In this context, Information Gain helps select the most informative features by measuring how much each sensor feature reduces the uncertainty in the dataset. Features that provide high Information Gain are fed into classification algorithms, improving the accuracy and reliability of the model. Especially in e-nose systems where a large number of sensors and different gas components are used, the Information Gain method eliminates unnecessary or low-contribution variables and optimizes the classification process. Furthermore, this method enables faster model training and increases generalization ability. Information Gain analyses also guide e-nose design and sensor placement strategies, revealing which sensors capture diabetes markers more effectively, thus increasing system efficiency [40].

### 2.6. Cross Validation

In this study, a 5-fold (k = 5) cross-validation method is used to reliably evaluate model performance for diabetes detection using e-nose data. The dataset is divided into five subsets of approximately equal size, each used for testing, and the remaining four subsets for training. This approach measures the model’s performance on different data subsets and reduces the risk of overfitting. Thanks to 5-fold validation, performance metrics such as accuracy, precision, and sensitivity of classification algorithms are obtained more reliably. Furthermore, this method more objectively reveals the impact of sensor-based features on the model and ensures consistent performance across different groups of individuals. In conclusion, k = 5 Cross Validation plays a critical role in improving the accuracy and generalization ability of e-nose-based diabetes detection systems [41].

### 2.7. Confusion Matrix

**Figure 2:**
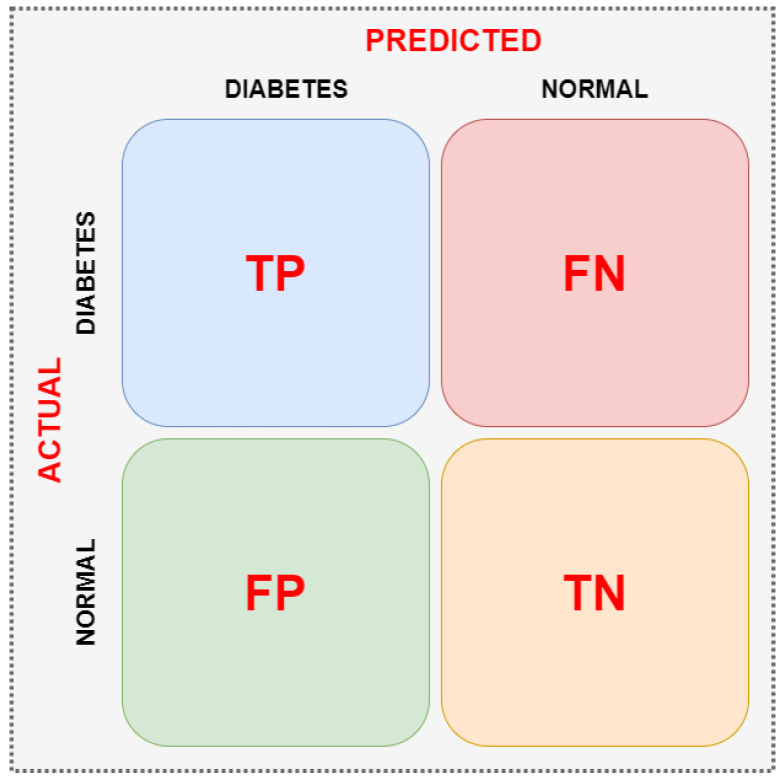
Two-class confusion matrix.

In this study, the Confusion Matrix method is applied to thoroughly evaluate the performance of classification models for diabetes detection using e-nose data. The Confusion Matrix visualizes the relationships between the model’s predicted classes and the actual classes and categorizes correct or incorrect classifications into four basic categories: True Positive (TP), False Positive (FP), True Negative (TN), and False Negative (FN). This demonstrates not only the overall accuracy but also the model’s success in distinguishing between diabetic and healthy individuals. The matrix indicates when classification algorithms make errors and in which classes they are strong, serving as a critical tool for model improvement and analyzing the effects of sensor data. The resulting Confusion Matrix results form the basis for calculating performance metrics (sensitivity, specificity, F1 score, etc.) [42]. An example confusion matrix is shown in Figure 2.

### 2.8. Performance Metrics

In this study, four key performance metrics were used to evaluate the success of diabetes classification models developed using e-nose data: Accuracy (%), F1 Score, Precision, and Recall. Accuracy (%) provides a quick overview of overall accuracy by showing the ratio of correctly classified examples to the total number of examples. Precision indicates how many of the model’s positive predictions for a given class are actually correct and focuses on minimizing false positives in correctly identifying individuals with diabetes. Recall measures how accurately the model can recognize true positive examples, ensuring that individuals with diabetes are not missed. The F1 Score, calculated as the harmonic mean of Precision and Recall, demonstrates the model’s balanced performance by accounting for inter-class imbalances; it is particularly important for evaluating diabetes classes with unequal distributions. Using these metrics together allows for a comprehensive assessment of not only the overall accuracy but also the model’s effectiveness in distinguishing between diabetic and healthy individuals [43]. Table 1 shows the formulas of the performance metrics used in the study.

## 3. Experimental Results

**Figure 3:**
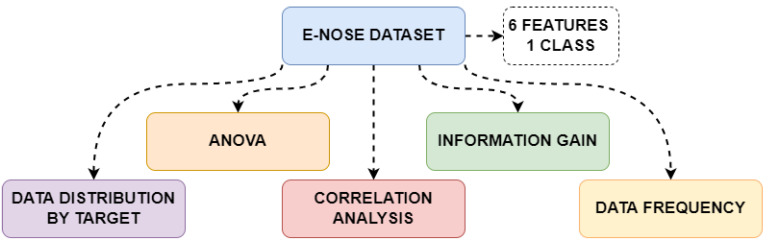
Data analysis methods used in the study.

In this study, machine learning methods were applied to determine whether individuals had diabetes or were healthy (normal) using breath sample data obtained from 1000 individuals via various sensors. Detailed analyses were performed on the measurements in the dataset. The study was conducted on a computer equipped with an Intel^®^ Core i7™ 12700K 3.61 GHz processor, an NVIDIA GeForce RTX 3080Ti graphics card, and 64 GB of RAM. The machine learning methods used are: ANN, DT, GB, NB, and AB. ANOVA and Information Gain methods were applied to determine which sensor datasets were more effective in class prediction. As a result of these analyses, the most effective features were selected and reclassification was performed using the same machine learning methods; the obtained results were compared with the classification results using all features. Correlation analysis was performed to determine the relationships between the sensor datasets. The frequency distributions of measurements from each sensor were examined, and data analysis was performed and interpreted based on their mean values. Furthermore, to reduce the data dimensionality, PCA was applied to perform classification operations on lower-dimensional data. The algorithms were coded using the Python programming language. Performance metrics for the machine learning algorithms used in the study are presented in Table 2. The data analysis steps performed in the study are shown in Figure 3.

**Figure 4:**
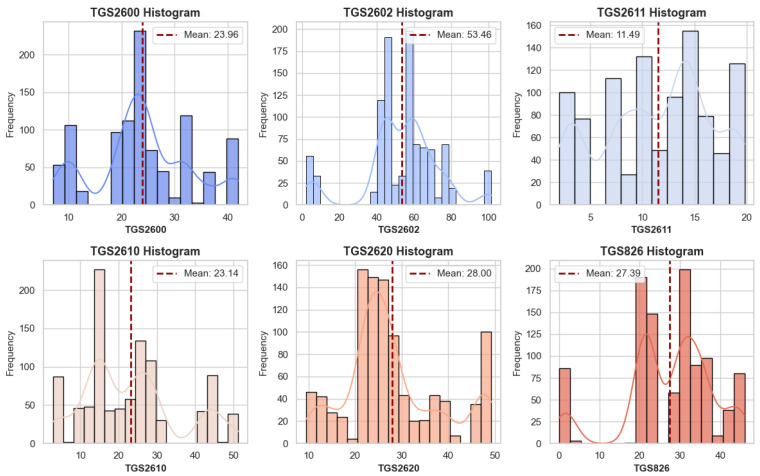
Frequency graphs of all features.

Before classifying e-nose data, which can be used in healthcare, food, agriculture, and defense, a detailed analysis of its consistency is necessary. Therefore, the study analyzed the data using five different methods. Six features in the dataset comprise data obtained from sensors. The names of the features are also represented by the sensor data. Data frequencies for each feature were determined to verify the consistency, data quality, and data balance of the data. Figure 4 presents frequency graphs for all features.

**Figure 5:**
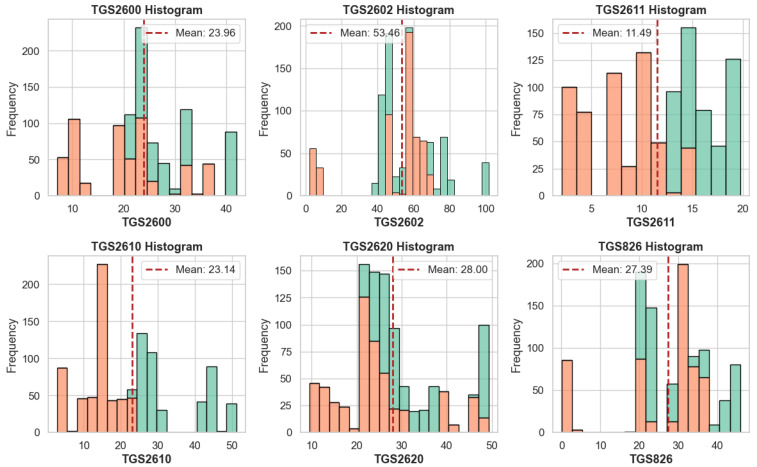
Distribution graphs of the sensor outputs by target.

As seen in Figure 4, the distribution of data obtained from the sensors varies. While the mean values also differ, the mean values for the TGS2610 and TGS2600 features are close to each other. However, an examination of the bar graphs reveals a discrepancy in the data for these two features. Consequently, the data for the features differs, and their use for classification appears to be unrelated. The distribution of feature data by class is shown in Figure 5.

**Figure 6:**
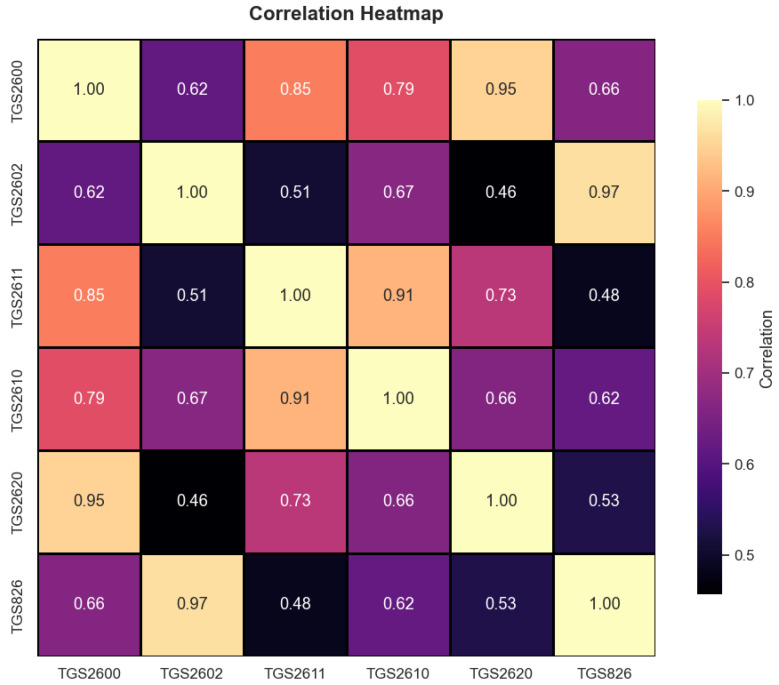
Correlation analysis of features.

As seen in Figure 5, the distribution of features by class is shown. It is clearly evident that the TGS2610 and TGS2611 features differ in predicting class. The graphs suggest that these features can play an active role in classification. Figure 6 shows the correlation analysis conducted to analyze the relationships between the features.

Figure 6 shows the relationship between the features. As the correlation value approaches 1, the relationship between the features increases. As it approaches 0, it decreases. According to Figure 6, it can be understood that the features are not independent of each other and that the features in the dataset are consistent with each other. This ensures that the features in the dataset are consistent with each other and that there is no classification problem. A correlation value of 1 indicates a linear relationship between the features, while a correlation value of 0 indicates either no relationship or an irregular relationship.

**Figure 7:**
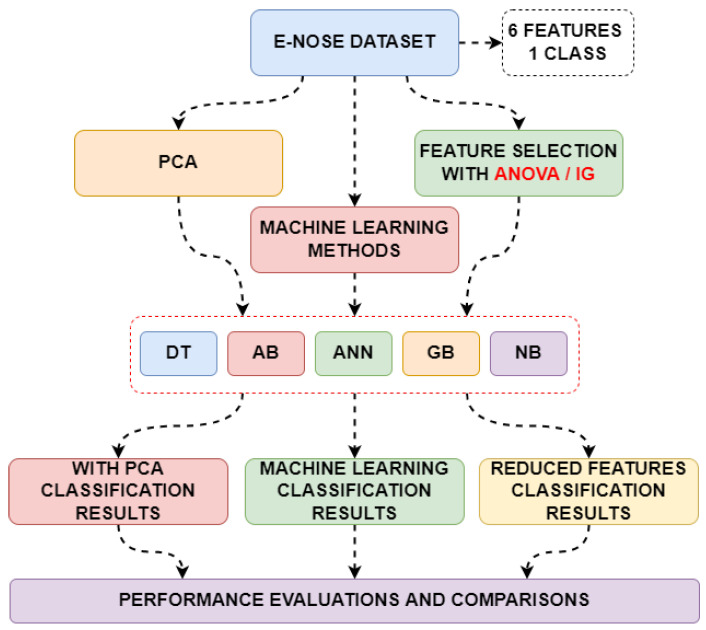
Classification stages of different variations in the dataset.

After detailed data analysis, the classification steps were carried out. In the classification steps, the data was first directly fed into the machine learning methods, and the class feature was estimated. In the second stage, the data size was reduced using the PCA method and fed back into the same machine learning methods. In the third stage, effective features were determined using ANOVA and Information Gain methods, and these features were fed back into the same machine learning methods. Performance metrics, training, and testing times obtained from all classifications were obtained and compared. The flow chart showing these processes is shown in Figure 7.

**Figure 8:**
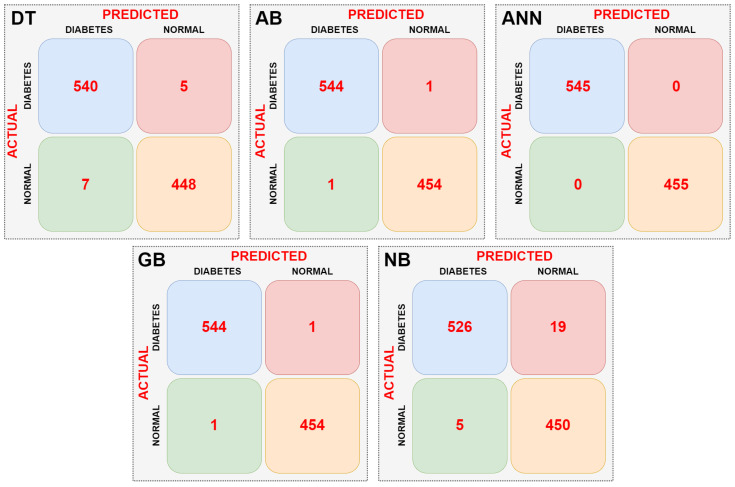
Confusion matrices obtained as a result of classifications made without data pre-processing.

In the first stage, the dataset was directly input to the DT, AB, ANN, GB, and NB machine learning models without any preprocessing, and the complexity matrix for each model was obtained. The resulting complexity matrices for each model are shown in Figure 8.

When the confusion matrices of the machine learning models are examined according to Figure 8, it can be seen that the ANN model is the most successful. The ANN model did not misclassify any data. The NB model had the lowest success rate. It incorrectly classified a total of 24 data points. The AB and GB models performed equally well. They correctly classified an equal number of data points. Performance metrics of the models were calculated using the confusion matrix data of the machine learning models. Table 3 shows the performance metrics of the machine learning models.

According to Table 3, the most successful model is the ANN model, with 100% success. The least successful model is the NB model, with 97.6%. When other performance metrics, excluding classification success, are examined, it is seen that these metrics are also achieved in parallel with the classification success.

**Figure 9:**
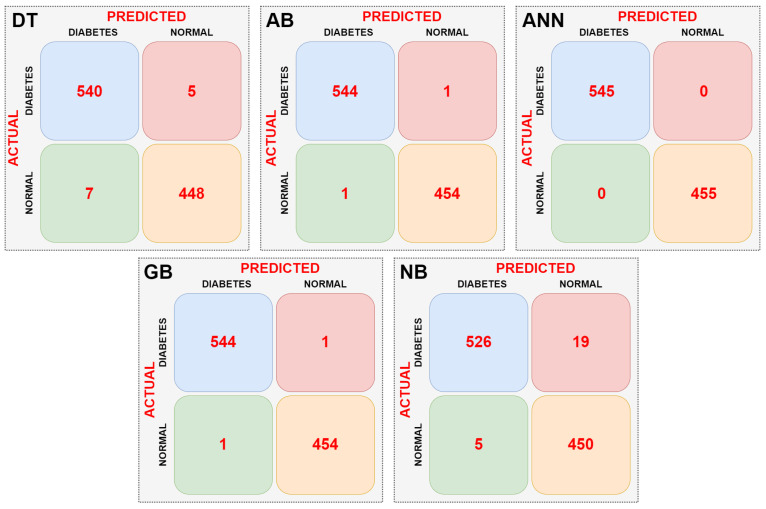
Confusion matrices obtained as a result of the classifications with PCA dimension reduction.

In the second stage, the dataset was subjected to dimension reduction using the PCA method. The data obtained from the PCA was then fed into the DT, AB, ANN, GB, and NB models. Confusion matrices were obtained for each machine learning model. The resulting confusion matrices are shown in Figure 9.

When the confusion matrices of the machine learning models are examined, as shown in Figure 9, it can be seen that the ANN model is the most successful. The ANN model did not misclassify any data. The NB model had the lowest success rate. It incorrectly classified a total of 24 data points. The AB and GB models had the same success rate. They correctly classified an equal number of data points. Performance metrics of the models were calculated using the confusion matrix data of the machine learning models. According to the data in the tables, it was observed that the performance of the models did not change in the classification performed with dimensionality reduction using PCA. Table 4 presents the performance metrics of the machine learning models.

According to Table 4, the most successful model is the ANN model, with 100% success. The lowest-performing model is the NB model, with 97.6%. When examining performance metrics other than classification success, it is seen that these metrics are also obtained in parallel with the classification success. While data reduction using PCA does not affect performance, it is believed to provide significant savings in training and testing time.

In the third stage, ANOVA and Information Gain methods were used to identify the features in the dataset that would be most effective in classification. The values obtained from the analysis performed with these methods are shown in Table 5.

**Figure 10:**
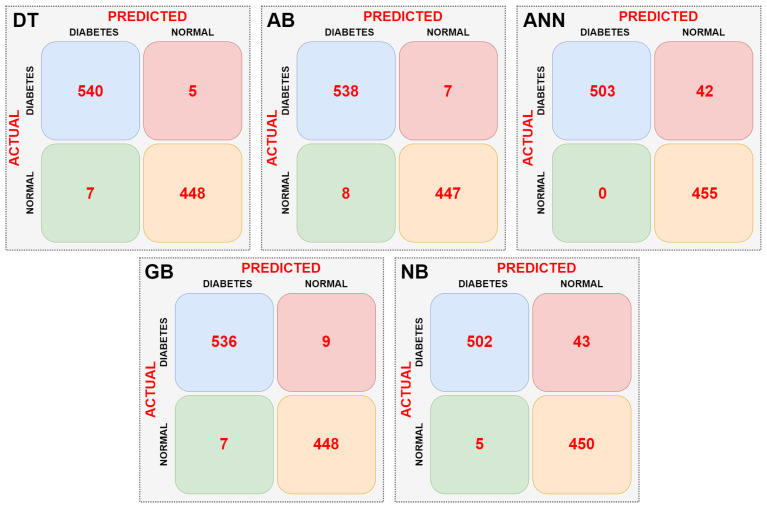
Confusion matrices obtained as a result of classifications made with 2 features.

As seen in Table 5, the data obtained from the ANOVA and Information Gain methods show that the TGS2610 and TGS2611 feature values are significantly higher than the other features. These two features are the most critical features that impact the success of machine learning methods. While they do not increase the success of classification models, they are expected to shorten the classification time. These two features were given as input to the DT, AB, ANN, GB, and NB models, and reclassification was performed. Confusion matrices were obtained for each model as a result of the classification. The resulting confusion matrices are shown in Figure 10.

Figure 10 shows that the most successful model is the DT model. The least successful model is the NB model. The values obtained from the classifications performed with the models using two features appear to be lower than those obtained without data preprocessing and with dimensionality reduction using PCA. However, it is believed that this will provide significant training and testing time savings. Performance metrics of the machine learning models were calculated using the data from the resulting complexity matrices. The resulting performance metrics are presented in Table 6.

According to Table 6, the most successful model is the DT model, with a 98.8% success rate. The lowest-performing model is the NB model, with a 95.2% success rate. When examining performance metrics other than classification success, it is seen that these metrics are also parallel to the classification success rate. The classification success rate of the DT model remained unchanged in the classification performed with two features. A decrease was observed in the other models.

The classification success rates and train/test time values of the models are shown in Table 7 for classification without data preprocessing, classification performed with dimensionality reduction using PCA, and classification performed with two features using feature selection using ANOVA/Information Gain methods.

Table 7 shows the effects of raw data, PCA, and only two features on five different classification models (DT, AB, ANN, GB, and NB). When raw data and PCA were used, the accuracy rates were largely maintained. The results remained almost unchanged in the DT, AB, ANN, and GB models. However, a decrease in accuracy was observed when only two features were used; this was particularly evident in the ANN, where the drop from 100 to 95.8% was significant. This suggests that excessive dimensionality reduction may limit the model’s learning capacity. An examination of training and testing times reveals that dimensionality reduction generally reduces processing times. For example, after PCA, the training time for AB decreased from 0.042 s to 0.032 s. However, the time advantage was more limited for complex models like ANN. NB performed consistently in terms of both accuracy and speed, with only minor improvements in time after dimensionality reduction. In general, dimensionality reduction with PCA can optimize training time without losing accuracy, while excessive feature reduction (two features) negatively impacted the performance of most models.

## 4. Conclusions

In this study, e-nose-based diabetes detection was performed using breath samples collected from 1000 individuals using different sensors. ANN, DT, GB, NB, and AB algorithms were applied in the study, and the effectiveness of sensor data in class prediction was evaluated using ANOVA and Information Gain methods. Furthermore, PCA was used to reduce the data dimensionality, and classification performance was compared across all features and selected critical features. The results revealed that the ANN model was the most successful with 100% accuracy, while the NB model performed the least with 97.6% accuracy. While dimensionality reduction using PCA did not significantly change accuracy values, significant improvements were achieved in training and testing times. ANOVA and Information Gain analyses revealed that the TGS2610 and TGS2611 sensors were the most effective in classification. Classifications using only these two features resulted in a decrease in accuracy, particularly in the ANN model. However, the classification time was significantly reduced in other models. The study’s results provide critical insights for e-nose-based diabetes detection, from sensor selection to algorithm optimization. The most effective sensors identified through ANOVA and Information Gain analysis enable shorter classification times and more efficient use of processing resources. Furthermore, comparing different classification algorithms on the same dataset provides an important reference for model performance in the literature. PCA, along with dimensionality reduction and feature selection, demonstrated that data size can be reduced while minimizing accuracy loss, thus demonstrating increased data efficiency in real-time applications. However, the study has several limitations. The dataset used covers only 1000 individuals, which may limit the generalizability of the model to larger and diverse populations. Furthermore, only certain types of sensors were used, and different sensor types or combinations may affect performance. Some models experienced a loss of accuracy when classifying with only critical features, indicating that excessive dimensionality reduction may limit the learning capacity of complex models. Future studies are recommended to expand the dataset and increase model generalizability with participants from diverse geographic regions. Furthermore, increasing the variety of sensors and utilizing hybrid models can further optimize accuracy and speed. AI-powered e-nose systems may be used as non-invasive tools in clinical diagnostic processes in the future, making significant contributions to routine screening, early diagnosis, and patient monitoring. They could also be applied to the rapid detection of metabolic or chemical compounds in healthcare, food, agriculture, and defense. This study contributes significantly to the literature by providing both a methodological guide and a performance comparison for e-nose-based diabetes detection. The combination of machine learning algorithms and data analysis techniques stands out as a promising approach for developing early diagnosis and clinical decision support systems.

## Figures and Tables

**Table 1:** Performance metrics formulas.

**Metrics**	**Equation**
Accuracy	$\frac{TP+TN}{TP+TN+FP+FN}$
Precision	$\frac{TP}{TP+FP}$
Recall	$\frac{TP}{TP+FN}$
F-1 Score	2 × $\frac{Precision\;\times \;Recall}{Precision+Recall}$

**Table 2:** Parameters of machine learning models.

Models	Parameters
**Decision Tree (DT)**	Min. number of instances in leaves: 2
	Do split subsets smaller than: 5
	Limit the maximal tree depth to: 100
	Stop when majority reaches: 95%
**AdaBoost (AB)**	Base estimator: tree
	Number of estimators: 50
	Learning rate: 1
	Loss (regression): Linear
**Artificial Neural Network (ANN)**	Neurons in hidden layers: 100
	Activation: ReLu
	Solver: Adam
	Regularization: α = 0.0001
	Number of iterations: 200
**Gradient Boosting (GB)**	Number of trees: 100
	Learning rate: 0.1
	Limit depth of individual trees: 3
	Do split subsets smaller than: 2
	Fraction of training instances: 1
**Naive Bayes (NB)**	Alpha: 0.5
	Fit prior: True
	Class prior: [0.5, 0.5]
	Var smoothing: 1 × 10^−8^

**Table 3:** Performance metrics obtained from the machine learning models after data preprocessing classification.

	Accuracy (%)	F1 Score	Precision	Recall
**DT**	98.800	0.988	0.988	0.988
**AB**	99.800	0.998	0.998	0.998
**ANN**	100.00	1.000	1.000	1.000
**GB**	99.800	0.998	0.998	0.998
**NB**	97.600	0.976	0.976	0.976

**Table 4:** Performance metrics obtained from the machine learning models after classification using dimensionality reduction using PCA.

	Accuracy (%)	F1 Score	Precision	Recall
**DT**	98.800	0.988	0.988	0.988
**AB**	99.800	0.998	0.998	0.998
**ANN**	100.000	1.000	1.000	1.000
**GB**	99.800	0.998	0.998	0.998
**NB**	97.600	0.976	0.976	0.976

**Table 5:** ANOVA and information gain analysis results.

	ANOVA	Information Gain
**TGS2610**	1855.695	0.824
**TGS2611**	1853.061	0.803
**TGS2600**	318.420	0.274
**TGS2620**	147.882	0.234
**TGS2602**	59.420	0.274
**TGS826**	44.452	0.296

**Table 6:** Performance metrics obtained from the machine learning models after classification using the TGS2610 and TGS2611 features.

	Accuracy (%)	F1 Score	Precision	Recall
**DT**	98.800	0.988	0.988	0.988
**AB**	98.500	0.985	0.985	0.985
**ANN**	95.800	0.958	0.962	0.958
**GB**	98.400	0.984	0.984	0.984
**NB**	95.200	0.952	0.955	0.952

**Table 7:** Classification success rates and train/test time values for all classification operations.

				(Raw Data)		PCA		2 Features	
	Accuracy (Raw Data)	Accuracy (PCA)	Accuracy (2 Features)	Train Time (s)	Test Time (s)	Train Time (s)	Test Time (s)	Train Time (s)	Test Time (s)
**DT**	98.800	98.800	98.800	0.007	0.001	0.011	0.002	0.012	0.001
**AB**	99.800	99.800	98.500	0.042	0.017	0.032	0.008	0.027	0.002
**ANN**	100.000	100.00	95.800	1.375	0.018	1.659	0.005	1.713	0.001
**GB**	99.800	99.800	98.400	0.985	0.002	0.729	0.006	0.686	0.004
**NB**	97.600	97.600	95.200	0.024	0.017	0.012	0.006	0.023	0.002

## Data Availability

The dataset downloaded from https://www.kaggle.com/datasets/muhammadrizwan111/enose-sensor-dataset-for-predicting-human-diseases (Access date: 10 July 2025).
